# Antibiotic Resistance in *Escherichia coli* from Pigs in Organic and Conventional Farming in Four European Countries

**DOI:** 10.1371/journal.pone.0157049

**Published:** 2016-06-30

**Authors:** Julia Österberg, Anne Wingstrand, Annette Nygaard Jensen, Annaelle Kerouanton, Veronica Cibin, Lisa Barco, Martine Denis, Sören Aabo, Björn Bengtsson

**Affiliations:** 1 National Veterinary Institute (SVA), Uppsala, Sweden; 2 National Food Institute, Technical University of Denmark (DTU), Copenhagen, Denmark; 3 French Agency for Food, Environment and Occupational Health Safety (ANSES), Ploufragan, France; 4 Istituto Zooprofilattico Sperimentale delle Venezie (IZSVe), Legnaro, Italy; Leiden University, NETHERLANDS

## Abstract

Organic pig production differs in many ways from conventional production of pigs, e.g., in antibiotic use, herd structure, feeding regimes, access to outdoor areas and space allowance per pig. This study investigated if these differences result in a lower occurrence of antibiotic resistance in organic slaughter pigs in Denmark, France, Italy and Sweden. Samples were taken from the colon content and/or faeces and minimum inhibitory concentrations (MIC) of ten antibiotics were determined in isolates of *Escherichia coli*. In addition, the proportion of tetracycline (TET) resistant *E*. *coli* in colon content and/or faeces from individual pigs was determined. In all four countries the percentage resistance to ampicillin, streptomycin, sulphonamides or trimethoprim was significantly lower in *E*. *coli* from organic pigs. In France and Italy, the percentage of isolates resistant to chloramphenicol, ciprofloxacin, nalidixic acid or gentamicin was also significantly lower in the *E*. *coli* from organic pigs. Resistance to cefotaxime, was not found in any country. The percentage of *E*. *coli* isolates resistant to TET as well as the proportion of TET-resistant *E*. *coli* was significantly lower in organic than in conventional pigs, except in Sweden where TET-resistance was equally low in both production types. There were also differences between countries within production type in the percentage resistance to individual antibiotics as well as the proportion of TET-resistant *E*. *coli* with lower median proportions in Sweden and Denmark compared to France and Italy. The study shows that in each of the four countries resistance in intestinal *E*. *coli* was less common in organic than in conventional pigs, but that there were also large differences in resistance between countries within each production type, indicating that both country- and production-specific factors influence the occurrence of resistance.

## Introduction

Antibiotic resistance undermines the possibility to effectively treat bacterial diseases in humans and animals and it is one of the major global threats for the future [1]. The main driver of resistance is the use of antibiotics in combination with inadequate measures to control spread of resistant bacteria. Use of antibiotics causes a selection pressure favouring resistant bacteria and their spread in bacterial populations harboured by humans and animals and in the environment [1, 2].

The presence of resistant bacteria among animals is worrying from a veterinary clinical perspective but the zoonotic aspect is also of concern. Resistant bacteria that emerge among food-producing animals can spread to humans, for example, along the food production chain [3, 4]. To raise food-producing animals without extensive use of antibiotics is therefore a global high priority issue [5, 6]. MIC testing of commensal intestinal *E*. *coli* from healthy animals is commonly used as indicator for the occurrence of resistance in animal populations [7] and forms the basis for mandatory monitoring of production animals in EU (EU Commission directive 2003/99/EU). European monitoring activities show that there are large differences between countries in antibiotic resistance [8] and use [9]. Although data from the different countries in these studies may not be fully comparable, a positive association between use of antibiotics and resistance in *Escherichia coli* from healthy food-producing animals is indicated [10–12].

In organic food animal production in the EU, several chemical substances and preventive medications are prohibited due to a generally restrictive principle against external inputs [5]. Due to this, the use of antibiotics in organic food animal production is restricted and the withdrawal period before slaughter for human consumption is longer. Although the use of antibiotics in pig production is the likely main driver for occurrence of antibiotic resistance in pigs, it may not exclusively determine the level of resistance in organic and conventional pig herds. Factors such as herd size, animal contacts, feeding regimes, outdoor access and space allowance per pig could also influence emergence and persistence of resistant bacteria. Studies that explore differences in resistance between farms and production types, for example organic and conventional, can provide information about what practices reduce antibiotic resistance [13, 14].

Most studies comparing resistance in organic and conventional production have been performed regionally and/or mainly studied resistance in zoonotic bacteria such as *Salmonella* and *Campylobacter* [14, 15]. To explore resistance in commensal intestinal *E*. *coli* from healthy pigs in organic and conventional production in different regions could be one way to gain basic knowledge for further studies aiming to identify risk factors for antibiotic resistance in pig production. Thus, the aim of the present study was to compare the occurrence of antibiotic resistance in slaughter pigs from conventional and organic production in four European countries.

## Materials and Methods

The study was conducted according to relevant national and international guidelines. Field samples were faecal samples (only) collected at pig farms in two of the participating countries (Italy and Sweden) with the permission of the owner of the pig herd. According to national legislation in Sweden (SJVFS 2015:24) and Italy no ethical permission is required when the animals are not subject to any manipulation and when the owner of the animals has given his/her permission.

### Sampling of colon contents and faeces

Samples was taken of colon contents from healthy slaughter pigs at abattoirs slaughtering both organic and conventional pigs in Denmark, France and Sweden and of faeces from healthy pigs close to slaughter weight at farms in Italy and Sweden. The goal was to include two pigs from each of 25 herds of each production type (organic and conventional) from each country. In Italy an additional three pigs were sampled on each farm. An overview of the final sampling procedure in each country is presented in Table 1. After evisceration at abattoirs, contents from the mid-segment of colon were collected into sterile plastic cups. At farm, rectal faecal samples were collected from individual pigs. Samples were kept refrigerated until analysis within one to four days after sampling in respective country.

**Table 1:** Overview of sampling of pigs from conventional (Con) and organic (Org) herds in abattoirs and/or herds in each country.

Country	Herds		Animals		Sampling locations		Sampling period
	(number)		(number)		(number)		
	Org	Con	Org	Con	Abattoir	Herd	
Denmark	25*	26	52	52	1	-	October—2012-September 2013
France	25	25	50	50	1	-	April-October 2012
Sweden	18	18	36	36	4	-	August 2012-October 2013
Sweden	18	18	36	36	-	36	August 2012-October 2013
Italy	25	25	125	125	-	50	November2012- March 2013

* one herd was sampled twice, on two different occasions.

### Laboratory analyses

To ensure comparability in MIC testing, we followed the guidelines for methodology and interpretation issued by the EU for mandatory monitoring of *E*. *coli* from healthy animals (Commission decision 2013/652/EU). However, MIC testing of a single or few isolates provides a limited knowledge on the actual resistance burden carried by animals. Therefore, we also quantified and compared the relative occurrence of resistance in individual pigs between each production type and between countries by determining the proportion of tetracycline (TET) resistant *E*. *coli* in intestinal content/faeces of individual pigs. Tetracycline is the most used antibiotic in pig production and TET-resistance was chosen to ensure sufficient data in the study of proportions.

#### Isolation and enumeration of total Escherichia coli and TET-resistant E. coli

In all four countries *E*. *coli* for susceptibility testing was isolated from intestinal content/faeces from two pigs from each farm by culture on 3MPetrifilm^™^ Select E. coli Count Plates (SEC plates 3M, St. Paul, MN, USA). In addition, total *E*. *coli* and TET-resistant *E*. *coli* in samples of colon contents or faeces were enumerated by plate counting using SEC-plates. In brief, the sample material was diluted 1:10 in peptone salt water (PSW) and homogenized, after which a ten-fold dilution series in PSW was prepared. Following the manufacturer’s instruction, 1mL from selected dilutions was added onto SEC plates. For a selection of sample dilutions, 1 mL was also mixed with 50 μL of a tetracycline solution (1344 mg/L) and added to SEC plates to a final concentration of 64 mg/L. Thereafter the plates were incubated in 44°C for 24 hours. Previous work has shown that this procedure allows growth only of tetracycline resistant *E*. *coli*, i.e. with MIC > 8g/mL [16].

Pure cultures of isolates of *E*. *coli* in Denmark, Italy, Sweden and France for susceptibility testing were obtained by streaking one or two colonies with blue-green colour from SEC plates without tetracycline onto blood agar plates followed by incubation at 37°C for 24 hours. Isolates were subsequently confirmed as *E*. *coli* by positive production of tryptophanase using the spot indole test and stored at -20°C pending analysis for antibiotic susceptibility.

In Italy, *E*. *coli* for susceptibility testing was also isolated from an additional three pigs from each farm using a different cultivation method. From these additional pigs, 1 g of faeces was homogenized in 9 mL of TSB (Tryptone Soya Broth) and then plated onto MacConkey agar, followed by incubation for 24 hours at 37°C. A single *E*. *coli* colony was then selected from the plate, biochemically confirmed as above and stored at -20°C pending analysis for antibiotic susceptibility.

#### Calculation of the estimated proportion of TET-resistant *E*. *coli* in colon and faecal samples

The estimated proportion of TET-resistant *E*. *coli* was calculated according the following equation:

$$Prop\;TET\;E.\;coli\;=\;\frac{Conc\;TET\;E.\;coli}{Conc\;E.\;coli}$$

To reduce uncertainty of proportions, only *E*. *coli* counts ≥ 4 CFU in the denominator were considered for the calculations.

In Sweden, the estimated proportion was not calculated in 12 samples from conventional and in 4 samples from organic pigs due to a laboratory error. In France, the percentage was not calculated in one sample from an organic pig due to counts below the criteria for calculation (< 4 CFU).

#### Antibiotic susceptibility testing by broth microdilution

In Denmark, Sweden and Italy, one isolate from each sample was tested for antibiotic susceptibility and in France, two isolates. Antibiotic susceptibility was determined from the minimum inhibitory concentration (MIC) using broth microdilution following the standards of the Clinical and Laboratory Standards Institute [17] and VetMIC panels (SVA, Uppsala, Sweden) (Sweden) or TREK-panels (TREK Diagnostic Systems LTD, East Grinstead, UK) (Denmark, France, Italy). *Escherichia coli* ATCC 25922 was used for quality control. To further ensure the comparability of MIC determinations, the participating laboratories took part in the yearly proficiency test organized by the EU Reference Laboratory—Antimicrobial Resistance (DTU, Copenhagen) in June 2012 shortly before the laboratory work in this study commenced. All laboratories performed satisfactorily in the test. The test included determination of MIC, by microdilution, of eight *E*. *coli* strains to a panel of antibiotics; this panel included the antibiotics in the present study.

Antibiotics tested were: ampicillin, cefotaxime, chloramphenicol, ciprofloxacin, gentamicin, nalidixic acid, streptomycin, sulphonamides, tetracycline and trimethoprim. In France, trimethoprim and sulphonamide were not tested separately but in combination. MICs were evaluated by epidemiological cut-off values (ECOFF) (Table 2) issued by EUCAST (www.eucast.org) and isolates of the non-wild type were considered microbiologically resistant, for brevity hereafter referred to as resistant.

**Table 2:** Frequency of resistance (percent) to selected antibiotics in *Escherichia coli* from slaughter pigs in Denmark, France, Italy and Sweden and univariable association between conventional (Con) vs. organic (Org) herds. Univariate odds ratios (OR), 95% confidence interval for OR (95% CI) and p-values for the association. n = number of isolates, nd = not done. Interpretive criteria for MICs (ECOFF) separating wild-type from non-wild type isolates are indicated (mg/L).

Antibiotic (ECOFF)	Denmark				France				Italy				Sweden			
	Con (n = 52)	Org (n = 52)	OR (95% CI)	p	Con (n = 94^a^)	Org (n = 100)	OR (95% CI)	p	Con (n = 125)	Org (n = 125)	OR (95% CI)	p	Con (n = 71^b^)	Org (n = 71^b^)	OR (95% CI)	p
Ampicillin (>8)	25.0%	3.9%	8.2 (1.7–39.9)	0.009	14.9%	13.0%	1.2 (0.4–3.2)	0.772	62.4%	9.6%	15.6 (6.5–37.9)	<0.001	18.3%	4.2%	5.1 (1.4–18.2)	0.013
Cefotaxime (>0.25)	0.0%	0.0%	nd		0.0%	0.0%	nd		0.0%	0.0%	nd		0.0%	0.0%	nd	
Chloramphenicol (>32)	0.0%	3.9%	nd		17.0%	1.0%	20.6 (2.6–161.4)	0.004	30.4%	2.4%	17.8 (5.4–58.4)	<0.001	1.4%	1.4%	nd	
Ciprofloxacin (>0.06)	0.0%	0.0%	nd		4.3%	1.0%	3.4 (0.5–37.9)	0.178	12.0%	0.8%	16.9 (2.2–128.1)	0.006	1.4%	1.4%	nd	
Gentamicin (>2)	5.8%	0.0%	nd		7.5%	6.0%	1.3 (0.5–3.5)	0.621	6.4%	0.8%	8.5 (1.01–71.3)	0.049	1.4%	0.0%	nd	
Nalidixic acid (>16)	0.0%	0.0%	nd		4.3%	2.0%	2.2 (0.4–11.3)	0.357	10.4%	0.8%	14.4 (1.9–110.4)	0.010	1.4%	0.0%	nd	
Streptomycin (>16)	44.2%	13.5%	5.1 (1.9–13.5)	0.001	66.0%	32.0%	4.1 (2.2–7.9)	<0.001	61.6%	9.6%	15.1 (6.8–33.4)	<0.001	25.4%	11.3%	2.7 (1.0–7.5)	0.054
Sulphonamides (>64)	24.6%	9.6%	5.0 (1.8–14.0)	0.003	nd	nd	nd		61.6%	15.2%	8.9 (3.9–20.6)	<0.001	25.4%	8.5%	3.7 (1.3–10.7)	0.016
Tetracycline (>8)	42.3%	13.5%	4.8 (1.5–15.5)	0.008	74.5%	46.0%	3.4 (1.7–6.7)	<0.001	74.4%	34.4%	5.5 (2.6–11.9)	<0.001	14.1%	9.9%	1.5 (0.5–4.5)	0.471
Trimethoprim (>2)	23.1%	7.7%	3.7 (1.1–12.4)	0.034	nd	nd	nd		50.4%	9.6%	9.6 (3.3–27.8)	<0.001	19.7%	1.4%	17.2 (2.2–132.2)	0.006
Trimethoprim & Sulphonamide (>1)	nd	nd	nd		40.4%	10.0%	6.1 (2.9–13.0)	<0.001	nd	nd	nd		nd	nd	nd	

^a^ Only one *E*. *coli* was tested in six samples;

^b^ No *E*. *coli* were isolated in one sample from organic and in one sample from conventional pigs.

### Statistical analyses

#### MIC

Differences between the occurrence of antimicrobial resistance (MIC) for the ten antibiotics in *E*. *coli* isolates from pigs in conventional and organic herds were analysed and compared by logistic regression analysis (Proc Genmod, SAS^®^ version 9.4, SAS Institute Inc., Cary, NC, USA). The outcome variable was antibiotic sensitivity (resistant or sensitive) and the explanatory variables were herd type (conventional or organic) and country (Denmark, France, Italy or Sweden). Swedish samples were stratified by sample type (colon contents or faeces). The resistance differences were expressed as Odds Ratios (OR). Repeated sampling in the herds was handled by applying a herd-level repeated statement to the model (correlation structure: compound symmetry). Initial univariate regression analysis of the effect of herd type and country on resistance was followed by multivariable analysis with both variables in the model. Initial univariate and multivariable analyses, did not find any significant difference in antibiotic resistance in the Swedish colon and faecal isolates (p>0.05), so these were grouped in the final models. Finally, an investigation of interaction between country and herd type was conducted. Due to significant interaction terms between herd type and country for all antibiotics except cefotaxime, presentation of country specific ORs for each antibiotic was chosen with a 95% confidence interval for the ORs and a significance level (p-value) for ORs being different from 1 (Table 1). ORs with p<0.05 were considered statistically significant.

#### Proportion of tetracycline resistant *E*. *coli*

**Fig 1:**
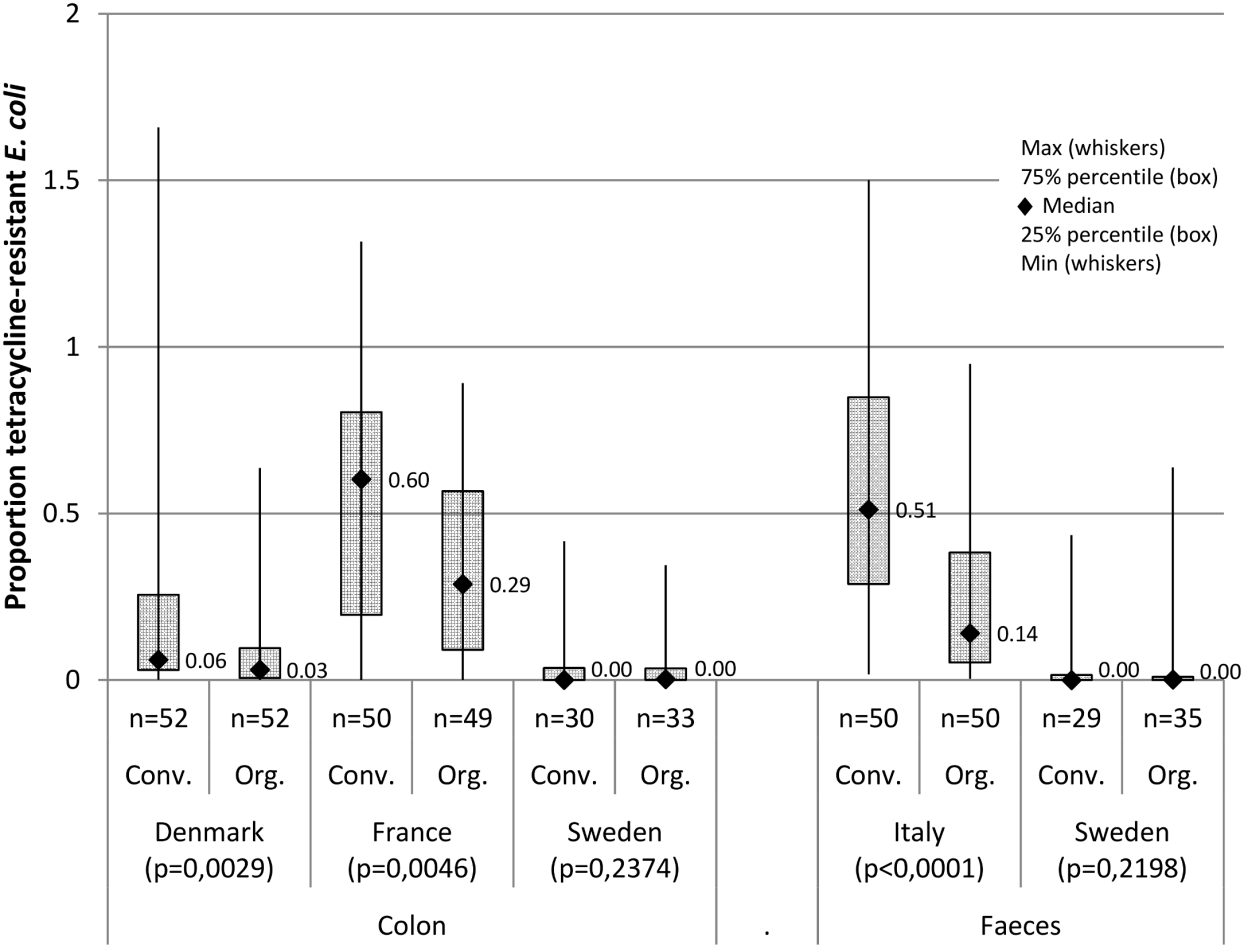
The distribution of the proportion of tetracycline resistant *E*. *coli* in samples of colon contents or faeces from 430 pigs in 110 conventional (Conv.) and 93 organic (Org.) herds in four European countries (median, 25% and 75% percentiles (box), maximum and minimum (whiskers)). n = number of samples; p = the significance level for the difference between distributions in conventional and organic herds for each country and sample type (Kruskal-Wallis test for non-normal distributions).

The distribution of proportions for each herd type, country and sample type are presented as the median proportion, 25% and 75% percentiles and minimum and maximum proportion (Proc Means,SAS^®^ version 9.4, SAS Institute Inc., Cary, NC, USA) (Fig 1). This is because there were non-normal, strongly skewed distributions of the estimated proportions of TET-resistant *E*. *coli* and many samples with proportions = 0 particularly from Denmark and Sweden. The level of significance (p-value) was tested for the difference between distributions of Prop TET in conventional and organic herds for each country and sample type (Kruskal-Wallis test for non-normal distributions, Proc Npar1way, SAS^®^ version 9.4, SAS Institute Inc., Cary, NC, USA). The difference between distributions was considered statistically significant at p<0.05.

## Results

By MIC testing of isolates of *E*. *coli* from Petrifilms without tetracycline, the most common traits in both production types in all four countries were resistance to ampicillin, streptomycin, sulphonamide, tetracycline, and trimethoprim (Table 2). In the French and Italian conventional production, chloramphenicol resistance was also common (17% and 30%, respectively), while in Denmark and Sweden resistance to this antibiotic was detected with low frequency (range 0–4% for both herd types). Resistance to ciprofloxacin, gentamicin and nalidixic acid was uncommon relative to the other antibiotics in all four countries. No resistance to cefotaxime was detected in this study (Table 2).

There were statistically significant differences in resistance between isolates from conventional and organic pigs in each of the four countries (Table 2). The largest differences were detected in Italy, where the risk for resistance in isolates from conventional production was significantly higher than in organic production for all ten antibiotics except cefotaxime (OR range: 5.5–17.8). In Italy, the highest OR (17.8, p < 0.001) was for chloramphenicol. In France the highest risk (OR 20.6, p = 0.004) was also for chloramphenicol, while ORs for streptomycin, tetracycline and trimethoprim-sulphonamide were lower, but still statistically significant (OR range: 3.4–6.1). In Denmark, there was a significantly increased risk for resistance to ampicillin, streptomycin, sulphonamide, tetracycline or trimethoprim (OR range: 3.7–8.2) in isolates from conventional pigs, while in Swedish conventional pigs, it was significantly higher for ampicillin, sulphonamide and trimethoprim (OR range: 3.7–17.2). ORs for resistance to the low-prevalent resistance traits, i.e., chloramphenicol, ciprofloxacin, gentamicin and nalidixic acid, in isolates from Denmark and Sweden were not determined due to zero prevalence in either or both herd types or because the prevalence was the same for both.

The estimated proportion of TET-resistant *E*. *coli* in colon contents and faeces—based on quantitative cultures for intestinal *E*. *coli*—revealed similar differences in the median and distribution of the proportions between production types as well as between the countries (Fig 1). The median proportions were lower in Denmark and Sweden compared to those in France and Italy. In Denmark, France and Italy, the median proportions in organic pigs were lower than conventional pigs and the distributions of proportions varied significantly between production types; however, there were large variations between countries within production types (Fig 1).

## Discussion

In each of the four countries, there was less occurrence of resistance to individual antibiotics in the organic than the conventional production. The median proportion of TET-resistant *E*. *coli* was also lower in the intestinal flora of the organic pigs, except in Sweden where it was equally low in both production types. These findings are in general agreement with those of a meta-analysis on resistance in various types of bacteria from organic or conventional farm animals and food thereof [15]. Similar results are also reported in a Danish study of intestinal *E*. *coli* in pigs [18] and in a study of intestinal *E*. *coli* and enterococci from healthy pigs in New Zealand [19]. In organic dairy production there is also less resistance than in conventional production for both intestinal *E*. *coli* from calves [20], *Staphylococcus aureus* from dairy cows [21] and in both these bacterial species in beef [22]. Moreover, methicillin resistant *Staphylococcus aureus* (MRSA), which is widespread among pigs in several European countries, is less common on organic pig farms than conventional ones [23, 24].

The present study also revealed differences in antibiotic resistance among countries for the same production type. For some antibiotics the occurrence of resistance in isolates of *E*. *coli* from conventional pigs in one country was lower than in organic pigs in another country. This was also seen for the proportion of tetracycline resistant *E*. *coli*, i.e. the median proportion in Swedish conventional pigs was lower than for organic pigs in the other countries. Other studies have also found greater differences in resistance for *Staphylococcus aureus* between countries (USA and Denmark) than between production systems [21]. This indicates a complex causality and a possible interaction of factors that determine the magnitude of emergence, the spread and the persistence of antibiotic resistance.

In a separate study the samples of intestinal content and faeces collected in the present study were analysed for sulphonamide, tetracycline, streptomycin and chloramphenicol resistance genes using real-time PCR quantification [25]. The differences in phenotypic resistance of *E*. *coli* between countries observed in the present study were corroborated by the detection of resistance genes in that study. The difference between organic and conventional pig production could however not be verified, possibly due to the limited discriminatory power of the by PCR quantification as suggested by the authors.

The approach of comparing the quantitative carriage of TET-resistance showed for the first time that some resistances can be carried by all animals but that there is a difference in the load of intestinal carriage between organic and conventional pigs. This quantitative analysis of antibiotic resistance, here shown for TET-resistance, may provide a basis for a more accurate principle for estimating occurrence of resistance than can be deducted from the MIC determinations. A similar quantitative approach, employing RT-qPCR, for detection of tetracycline resistant *Enterobacteriaceae* in meat was developed and used by Guarddon et al. [26]. These types of data are essential for the understanding of quantitative contamination of meat at slaughter and forms a better basis for quantifying consumer exposure to antibiotic resistance from pork than can be extracted from traditional MIC testing.

Resistance to chloramphenicol was common among isolates from conventional pigs in France and Italy but rare in Denmark and Sweden; this corroborates the EFSA data [8, 27, 28]. Chloramphenicol is banned for use in food-producing animals in EU and has not been used for many years in pig production in Europe. Resistance to chloramphenicol is therefore most likely due to co-selection through use of other antibiotics than chloramphenicol, as previously suggested [29, 30]. The much lower occurrence of chloramphenicol resistance in organic than conventional production in both countries warrants further study.

No intestinal *E*. *coli* isolate was resistant to the cephalosporin cefotaxime. This indicates that transferable resistance to third-generation cephalosporins by production of extended spectrum beta-lactamases (ESBL) or plasmidic AmpC beta-lactamases is not prevalent among *E*. *coli* in pigs in the four countries. This is in agreement with findings in the mandatory monitoring in EU 2011, where cefotaxime resistance occurred in 1.7% of 2337 isolates of *E*. *coli* from slaughter pigs from 10 member states [27]. However, this does not preclude that a minority of *E*. *coli* in the intestinal flora of pigs is cephalosporin resistant, the true extent of such low-prevalent resistance may therefore not be established, unless selective culture for cefotaxime resistant strains is used.

Use of antibiotics in the sampled herds was not investigated in this study. However, in the EMA monitoring of antibiotic sales 2012, Italy and France reported the largest overall sales—341.0 and 102.6 mg/PCU (population correction unit) respectively—whereas Denmark reported 44.1 mg/PCU and Sweden 13,5 mg/PCU [9]. Since these data are not based on sales of antibiotics specifically for pigs, they should be used cautiously [31]. Although a difference in antibiotic exposure in conventional herds is a likely explanation for the resistance differences between countries, detailed data on the use at herd level are needed to confirm this hypothesis. Correspondingly, the lower occurrence of resistance in organic herds suggests a lower use of antibiotics in organic than in conventional herds within the same country, as reported in a Danish study [18].

However, the factors behind the emergence and spread of resistant bacteria are more complex than direct selection pressure from antibiotic use. There is also co-selection whereby use of one antibiotic selects for resistance to other substances. Resistance is also augmented by transmission of resistant bacteria between individuals and bacterial ecosystems and by transfer of genetic elements between bacteria [2].

The prevalence of infectious diseases could explain differences in antibiotic use and subsequently the occurrence of resistance. A high occurrence of infectious diseases on a regional level implies a greater need to prevent and treat the infections. Moreover, the disease burden on a farm is influenced by animal management and disease control, herd size, level of biosecurity and hygiene [32, 33]. These factors may differ between herds and countries and therefore indirectly influence the occurrence of resistance. In addition, the structure of the production, for example, how pigs are moved and mixed with other pigs, is important in the spread of pathogens and resistant bacteria [34, 35]. Limited transfer of pigs between herds reduces the spread of infectious diseases and presumably, resistant bacteria. In this context, it is probably a protective factor that only a minor part of the organic breeding stock are derived from conventional production.

The direct importance for human and animal health of the findings in this study cannot be assessed. However, a high occurrence of resistance in the intestinal flora of food-producing animals increase the risk that resistant bacteria are passed on in the food chain [3, 4]. This also implies the potential danger of transmission of resistance genes from animal bacteria to human bacteria [1]. More importantly our findings indicate that that there are differences in the factors determining emergence, spread and persistence of resistance between organic and conventional pig herds. Although the same regulations for the use of antibiotics in organic production are applied throughout EU, the differences suggest that there are also country-specific factors. Future studies in this field should be directed at identifying these factors in agreement with recommendations from WHO on the importance of monitoring resistance to ensure and identify appropriate actions to reduce the use of antibiotics in food producing animals [1].

One drawback of this study is that colon contents collected at slaughter were analysed in Denmark and France, whereas faeces collected on farms was analysed in Italy, and both these sample types in Sweden. However, since the mean proportions of TET-resistant *E*. *coli* were similar in the two matrices for pigs from Sweden we expect that the difference in sample material only puts a negligible bias on our data.

In conclusion, the study showed that there are significant differences in antibiotic resistance in organic and conventional pig production in each of the four countries and between countries. For all four countries, resistance was substantially lower in organic than conventional pigs. Although the same regulations for the use of antibiotics in organic production are applied throughout EU, the differences suggest that there could be country-specific differences in use of antibiotics and/or other factors that contribute to the emergence, spread and persistence of resistant bacteria. Future studies in this field should be directed at identifying these factors. This knowledge, together with a continued effort to improve animal health and thereby reduce the overall need for antibiotics, would be valuable to reduce antibiotic resistance without compromising animal welfare.

## Supporting Information

S1 Table Data on MIC-results of *E*. *coli* strains. (XLSX)

S2 Table Data on proportions of tetracycline resistant *E*. *coli*. (XLSX)

S3 Table Data on sampled pig herds. (XLSX)
